# Association between antihypertensive medication and the risk of urinary tract infection (UTI) of outpatients: a retrospective cohort study

**DOI:** 10.1007/s15010-022-01895-8

**Published:** 2022-07-29

**Authors:** Niklas Gremke, Karel Kostev, Matthias Kalder

**Affiliations:** 1 Department of Gynecology and Obstetrics, Philipps-University, Marburg, Germany; 2 Epidemiology, IQVIA, Frankfurt, Germany

**Keywords:** Antihypertensive medication, Arterial hypertension (AH), Antibiotic therapy, Urinary tract infections (UTI), Pharmacological side effects

## Abstract

**Purpose:**

The aim of this retrospective study was to investigate the impact of a broad spectrum of antihypertensive (AH) medications on urinary tract infections (UTI) of outpatients diagnosed in general practices in Germany.

**Methods:**

This study included a total of 367,960 patients aged ≥ 18 years newly a diagnosed with UTI in 1274 general practices in Germany between January 2010 and December 2019. The analysis was conducted for five groups representing five AH therapy classes (diuretics (DIU); beta blockers (BB); calcium channel blockers (CCB); ACE inhibitors (ACEi); angiotensin II receptor blockers (ARB)), each containing 73,592 patients. A Cox regression model was used to analyze the association between each antihypertensive drug class and UTI incidence as compared to all other antihypertensive drug classes (as a group).

**Results:**

The incidence of UTI diagnosis was slightly higher in patients treated with DIU (8.6%), followed by ACEi (8.1%), ARB (7.9%), and CCB (6.5%). Antibiotic therapy for UTI was given in 5.6% of DIU and 4.3% of CCB patients. The incidence of UTI and antibiotic therapy was much higher in women than in men across all therapy classes. No significant increase or decrease in UTI incidence or antibiotic therapy was observed in any of the AH therapy classes investigated.

**Conclusion:**

The present study did not identify a significant increase or decrease of UTI incidence or antibiotic therapy in patients treated with ACEi, ACB, CCB, beta blockers or diuretics. Across all AH classes studied, the incidence of UTI and antibiotic therapy was higher in women than in men, although not significantly.

## Introduction

Arterial hypertension (AH) is the most common preventable risk factor for cardiovascular disease (CVD) and chronic kidney disease (CKD) and is considered as the biggest single contributor to the global burden of disease and to global mortality with a total of 9.4 million deaths per year [1, 2]. The etiology of AH includes genetic predisposition, as well as a complex interplay of environmental and pathophysiological factors affecting multiple blood pressure (BP) regulation systems [3, 4]. One of the major BP regulation systems is the renin angiotensin aldosterone system (RAAS) which has a wide-ranging effect on BP control by regulating vascular tonicity as well as blood volume and, therefore, plays a pivotal role in AH pathogenesis [5, 6]. Based on this pathophysiological knowledge, a diverse subset of antihypertensive pharmacotherapeutics affecting the RAAS-System [e.g., ACE Inhibitors (ACEi) and Angiotensin II receptor blockers (ARB)] have been approved and have entered clinical practice in the past [7, 8]. However, clinicians are faced with many more classes of antihypertensive drugs, such as β-adrenoreceptor blockers (BB), calcium channel blockers (CCB), and different types of diuretics (DIU) [9, 10]. The World Health Organization (WHO) recently published a new guideline for the pharmacological treatment of AH in adults based on the data from 32 systematic reviews and pinpointed thiazide diuretics, ACEi/ARB’s, and long-acting dihydropyridine calcium channel blockers as first-line therapeutic agents. Especially in the treatment of patients with multimorbidity and frailty, these guidelines recommend clinical judgment because of potential risks arising from treatment side effects such as acute kidney injury, hyperkalemia, hypotension, and syncope which can result in hospital admission and reduced adherence of patients to antihypertensive medications [11, 12].

In view of these known side effects, the emergence of urinary tract infections (UTI) has become a subject of intense discussion in literature and has also repeatedly been identified as a possible side effect of antihypertensive therapy [13–15]. In general, UTIs are one of the most common diseases seen in general practitioner practices in Germany and are classified as either lower (confined to the bladder) or upper (pyelonephritis) and either uncomplicated (no relevant functional or anatomical anomalies in the urinary tract) or complicated (when those anomalies are present) [16, 17]. However, the evidence in the literature to date regarding the possible association between antihypertensive drugs and UTI is unsatisfying and contradictory. In this context, a population-based prescription sequence symmetry analysis conducted by Pouwels et al. showed a statistically significant increased risk of developing UTI upon ACEi initiation, whereas no association was found between beta blockers and UTI treatment.

Therefore, the aim of the present study is to analyze the incidence of UTI as a function of antihypertensive therapy and to reveal any potential association between a broad spectrum of antihypertensive medication and UTI incidence in outpatients with AH treated in general practices in Germany.

## Methods

### Database

This study was based on data from the Disease Analyzer database (IQVIA), which contains drug prescriptions, diagnoses, and basic medical and demographic data obtained directly and in anonymous format from computer systems used in the practices of general practitioners and specialists [18]. The database covers approximately 3% of all outpatient practices in Germany. Diagnoses (according to the International Classification of Diseases, 10^th^ revision [ICD-10]), prescriptions (according to the Anatomical Therapeutic Chemical [ATC] classification system), and the quality of reported data are monitored by IQVIA. In Germany, the sampling methods used to select physicians’ practices are appropriate for obtaining a representative database of general and specialized practices. It has previously been shown that the panel of practices included in the Disease Analyzer database is representative of general and specialized practices in Germany [18]. Finally, this database has already been used in previous studies focusing on antihypertensive therapy [19, 20] as well as urinary tract infections [21, 22].

### Study population

**Fig. 1:**
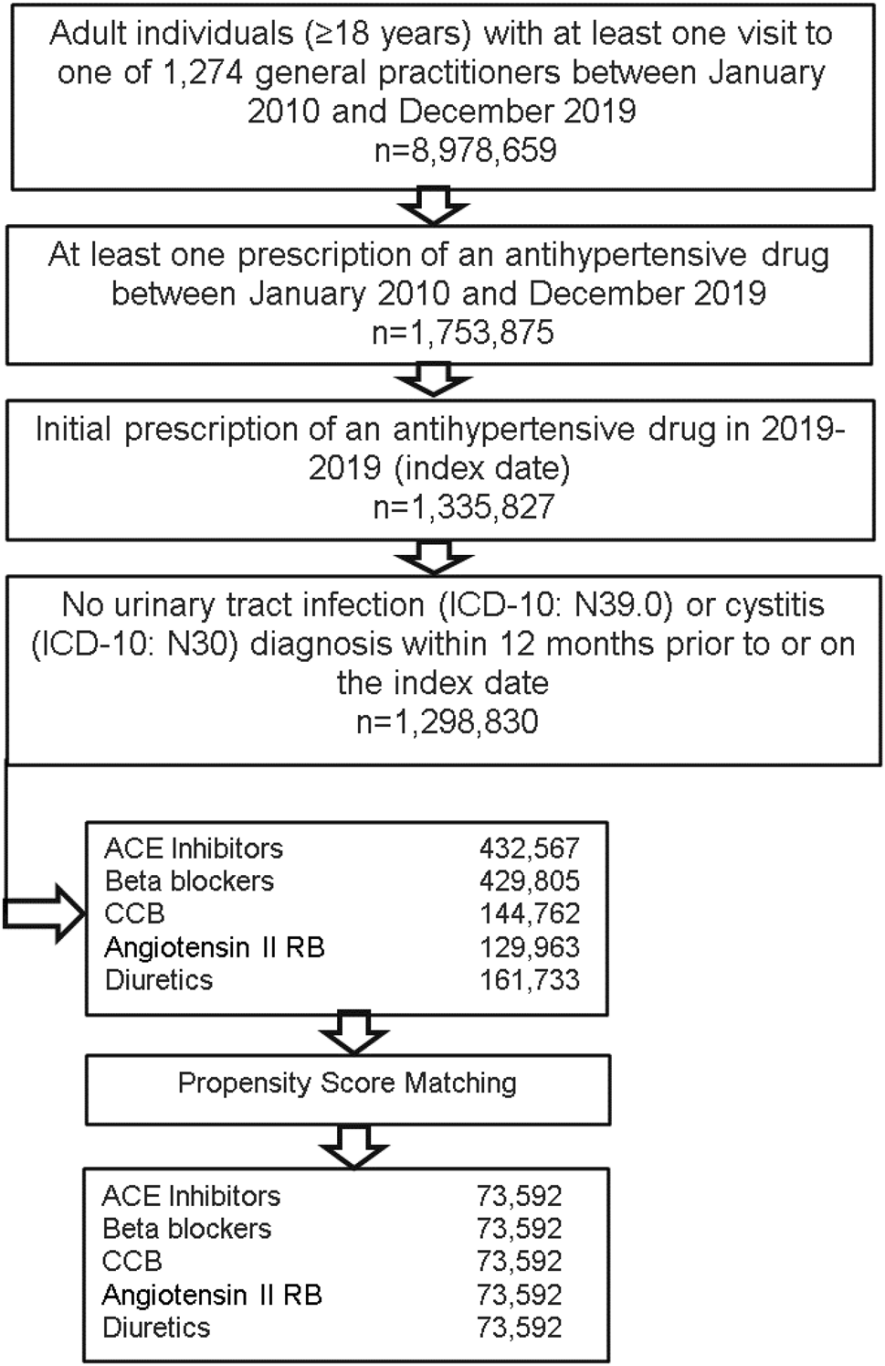
Selection of study patients

This retrospective cohort study included adult patients (≥ 18 years) with an initial prescription of a single antihypertensive therapy (diuretics, ATC: C03A; beta blockers, ATC: C07A; calcium channel blockers, ATC: C08A; ACE inhibitors, ATC: C09A; angiotensin II receptor blockers, ATC: C09A) in 1,274 general practices in Germany between January 2010 and December 2019 (index date; Fig. 1). Patients diagnosed with urinary tract infections, site not specified (ICD-10: N39.0) or acute cystitis (ICD-10: N30.0) within 12 months prior to or on the index date were excluded.

Five groups of patients treated with different antihypertensive drug classes were matched 1:1:1:1:1 to controls by propensity scores based on sex, age, and diagnoses documented within 12 months prior to or on the index date including diabetes (ICD-10: E10–E11), hypertension (ICD-10: I10), renal failure (ICD-10: N18, N19), and cancer (ICD-10: C00–C97), as well as therapy duration in months.

### Study outcomes and covariates

The main outcome of the study was the incidence of UTI within 12 months after the index date as a function of antihypertensive therapy. Each patient was followed for up to 12 months from the index date until the first UTI diagnosis was documented or antihypertensive therapy ended (either because of a switch to a different antihypertensive therapy or the addition of another drug class to the initial single therapy).

### Statistical analyses

Differences in the sample characteristics between five antihypertensive drug class groups were tested using chi-squared tests for categorical variables and Kruskal–Wallis tests for age. Conditional Cox regression models were applied to study the association between each antihypertensive drug class and UTI incidence as compared to all other antihypertensive drug classes (as a group). These models were applied separately for four age groups and also for women and men. As a sensitivity analysis, the outcome was additionally defined as the diagnosis of UTI plus a prescription of an antibiotic drug (ATC: J01) within 7 days following UTI diagnosis. Both regression models were adjusted for the physicians’ practices to reflect the diagnosis behavior of treating physicians. To counteract the problem of multiple comparisons and also due to the large patient samples, *p* values < 0.01 were considered statistically significant. Analyses were carried out using SAS version 9.4 (SAS institute, Cary, USA).

## Results

### Basic characteristics of the study sample

The present study included five therapy class groups, each comprising 73,592 patients (367,960 patients in total). The basic characteristics of the study patients are displayed in Table 1. Due to the matched pair design of this study, all five cohorts had the same age, sex, and comorbidity structure. The mean age [SD] was 66.5 [SD: 13.7] years; 55.2% of patients were women, prevalence of diabetes was 17.0%, hypertension 55.1%, renal failure 3.5%, cancer 5.9%. On average, patients were treated for approximately 250 days until therapy switch or break.

**Table 1:** Basic characteristics of the study sample after propensity score matching

Variable	Proportion affected among patients treated with ACE inhibitors (%)	Proportion affected among patients treated with beta blockers (%)	Proportion affected among patients treated with diuretics (%)	Proportion affected among patients treated with CCB (%)	Proportion affected among patients treated with ARB (%)	*P* value
N	73,592	73,592	73,592	73,592	73,592	
Age (mean, SD)	66.5 (13.7)	66.5 (13.7)	66.5 (13.7)	66.5 (13.7)	66.5 (13.7)	1.000
Age ≤ 60	32.5	32.5	32.5	32.5	32.5	1.000
Age 61–70	24.0	24.0	24.0	24.0	24.0	
Age 71–80	28.1	28.1	28.1	28.1	28.1	
Age > 80	15.5	15.5	15.5	15.5	15.5	
Female	55.2	55.2	55.2	55.2	55.2	1.000
Male	43.8	43.8	43.8	43.8	43.8	
Diabetes	17.0	17.0	17.0	17.0	17.0	1.000
Hypertension	55.1	55.1	55.1	55.1	55.1	1.000
Renal failure	3.5	3.5	3.5	3.5	3.5	1.000
Cancer	5.9	5.9	5.9	5.9	5.9	1.000
Therapy duration in days (mean, SD)	246 (153)	248 (151)	247 (150)	247 (152)	252 (147)	0.689

Proportions of patients given in % unless otherwise indicated

* SD* standard deviation

### Cumulative incidence of UTI diagnosis

**Fig. 2:**
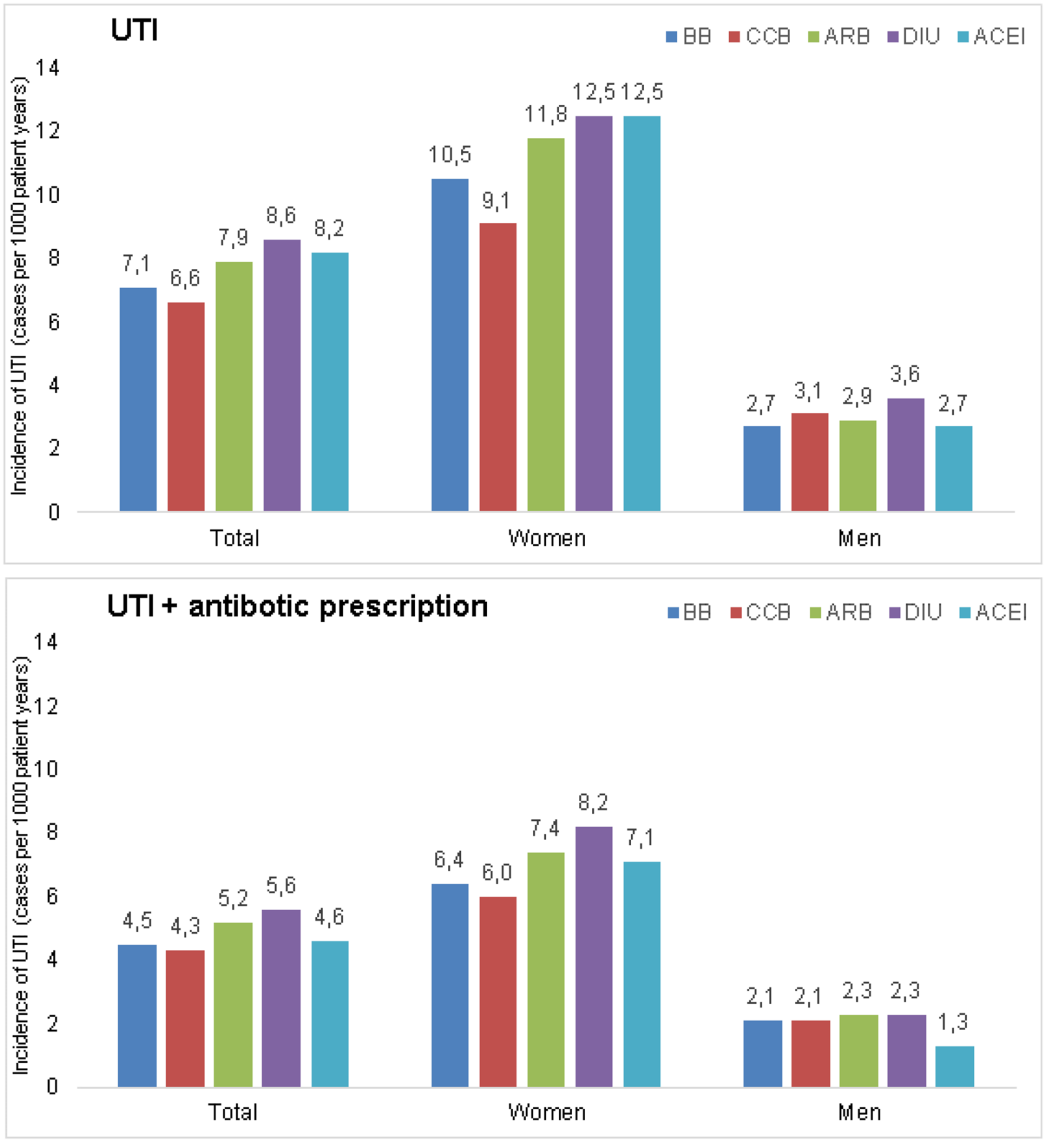
Proportion of individuals with a diagnosis of UTI and antibiotic therapy within 12 months after initiation of AH therapy in patients treated with different antihypertensive drugs

Figure 2 shows the incidence (cases per 1000 patient years) of UTI diagnosis. This incidence was slightly higher in patients treated with diuretics (8.6%), followed by ACEi (8.1%), and ARB (7.9%). The lowest incidence was in patients treated with CCB (6.5%). Antibiotic therapy for UTI was given in 5.6 DIU and 4.3% CCB patients. The incidence of UTI and antibiotic therapy was much higher in women than in men across all therapy classes.

### Association between antihypertensive therapy and incidence of UTI

Table 2 shows the results of the conditional regression analyses. No significant increase or decrease in UTI incidence or antibiotic therapy was observed in any of the therapy classes investigated. ARB was associated with an increased incidence of UTI (HR: 1.20, 95% CI 1.03–1.39) among women only with a *p* value of 0.020, but this was not considered statistically significant in this study.

**Table 2:** Association between antihypertensive therapy and the UTI diagnosis in patients followed in general practices in Germany (Cox regression models)

Cohort	BB vs. rest	CCB vs. rest	ARB vs. rest	DIU vs. rest	ACEI vs. rest
Total					
Diagnosis	0.94 (0.81–1.10)	0.92 (0.79–1.06)	1.13 (0.99–1.30)	1.04 (0.90–1.19)	0.97 (0.84–1.13)
Diagnosis + antibiotic prescription	1.00 (0.83–1.21)	0.99 (0.83–1.18)	0.97 (0.81–1.16)	1.06 (0.89–1.26)	0.98 (0.81–1.18)
Women					
Diagnosis	0.89 (0.75–1.07)	0.95 (0.81–1.11)	1.20 (1.03–1.39)	1.04 (0.89–1.21)	0.93 (0.79–1.09)
Diagnosis + antibiotic prescription	0.96 (0.80–1.18)	0.99 (0.81–1.21)	1.08 (0.88–1.31)	1.07 (0.88–1.29)	0.90 (0.73–1.12)
Men					
Diagnosis	1.18 (0.84–1.68)	0.82 (0.57–1.17)	0.94 (0.66–1.35)	1.06 (0.76–1.48)	1.05 (0.72–1.51)
Diagnosis + antibiotic prescription	1.18 (0.77–1.80)	0.98 (0.65–1.48)	0.64 (0.39–1.05)	1.01 (0.67–1.54)	1.27 (0.83–1.95)

## Discussion

The present retrospective cohort study included 73,592 patients for each class of antihypertensive drugs (367,960 patients in total) and found no significant increase or decrease in UTI incidence or antibiotic therapy in any of the therapy classes investigated. However, our results showed that the incidence of UTI and antibiotic therapy was much higher in women than in men across all antihypertensive therapy classes. These results are in line with the current literature, where UTI is considered the most common outpatient infection worldwide, having a prevalence of 23.3% for women and 6.8% for men, respectively [23]. A recently published study by Laupland et al. conducted population-based laboratory monitoring for all community-onset urinary tract infections among all residents of the Calgary Health region (~ 1.2 million residents) in Canada. During the 2 years of the study (2004–2005), there was a significant relationship between both age and sex and the incidence for outpatient onset UTI. The overall incidence in females was much higher than that in males (30.0 vs 5.0 per 1000, RR 5.98; 95% CI 5.81–6.15; *p* < 0.0001) and a substantial increase was observed in association with advancing age, reaching up to 637.8 (m) and 925.7 (f) per 1000/year in the very old (90 years of age) [24]. In most cases, UTI is treated with antibiotics, although focusing on more differentiated antibiotic drug prescription for UTI is an important issue with respect to reducing antimicrobial resistance. In this context a population-based cohort study recently showed that women suffering from UTI received significantly more antimicrobial drug prescriptions than men (45.2% vs. 12.6%) and the proportion of prescriptions for UTIs among all prescriptions with an indication code increased from 5.2% in 1996 to 14% in 2014 in men and from 28% in 1996 to 50% in 2014 in women [25]. Bearing in mind the issue of demographic change, the ageing of the population may partly explain the phenomenon of increased antibiotic drug prescriptions for UTI because antibiotics were most commonly prescribed to patients in older age categories [25, 26]. The difference observed between men and women is certainly also due to the anatomically determined length of the urethra, which means that UTIs occur less frequently in men.

However, UTIs are not only considered the most common of all bacterial infections but also cause enormous societal costs (cost of general and specialized medical visits, prescription drugs, diagnostic tests, hospitalizations etc.) [27–29]. In view of this, there is an urgent need to clarify the development of UTIs as possible side effects of different pharmacotherapeutics. Notably, the results of previous pharmacoepidemiologic studies on this topic are contradictory and insufficient. A prescription sequence symmetry analysis performed using the Dutch InterAction pharmacy prescription database, revealed a statistically significant increased risk of starting UTI antibiotic therapy after starting ACEi treatment, whereas no association was found between beta blockers and UTI treatment. Notably, the excess of patients who received an antibiotic prescription was only significant for the first month after ACEi initiation [13, 14]. Based on this finding, the authors introduced the hypothesis that ACEi may occasionally lead to a decreased glomerular filtration rate (GFR) and the risk of developing UTI may increase as a consequence of lower urine output. They also found that the association between ACEi therapy and UTI antibiotic prescription was stronger among patients with diabetes then those without diabetes, possibly due to diabetes-related renal impairment [13, 14, 30–33]. Nevertheless, this study is also subject to a number of methodological limitations (e.g., using nitrofurantoin prescriptions as a proxy with insufficient sensitivity for UTI) [14, 34].

Another study was conducted as a post hoc analysis using data from the REnal and Vascular ENdstage Disease Intervention Trial (PREVEND IT), a randomized, double-blind, placebo-controlled trial in which participants received pravastatin, fosinopril or placebo on a randomized basis in a 2 × 2 factorial design over 4 years [15, 35, 36]. In particular, the ACEi fosinopril was associated with an increased occurrence of initial UTI antibiotic prescriptions (HR, 1.82; 95% CI 1.16–2.88) [15]. However, this study was not designed and powered for a post hoc analysis and again used a proxy (antibiotic prescriptions) with limited sensitivity for the detection of UTI. In line with our observations, no studies are available that indicate that ACEi also increases the occurrence of UTI. Based on a reduced urine output as a result of ACEi treatment it could almost be assumed, that ACEi might increase the frequency of UTI [31, 32]. This hypothesis must be questioned, however, since a study has recently been published indicating that loop diuretics can deplete the renal cortico-medullary salt gradient as a major modulator of immune responses. As a result, renal transplant recipients suffer from a markedly increased rate of urinary tract infections (UTIs) upon treatment with diuresis-increasing loop diuretics [37].

Mansfield et al. analyzed the role of ACEi/ARBs or alternative AH therapeutics (β-blockers, calcium channel blockers, or thiazide diuretics) between April 1997 and March 2014 in a self-controlled case series (SCCS) in patients with acute kidney injury (AKI) following various infections (urinary tract infection, lower respiratory tract infection, and gastroenteritis). In this setting, acute infections (incl. UTI) are associated with a substantially increased risk of transient AKI among patients treated with AH drugs. However, the increase in relative risk was not higher among patients receiving ACEis/ARBs than in those treated with other AH drugs [38].

Unfortunately, there is still a lack of evidence in the literature for the incidence of UTI as a function of other AH therapeutics, e.g., CCB, ARBs, and diuretics. Particularly in view of this limited evidence, further research is needed concerning the role of UTI in patients receiving AH medications.

## Strengths and limitations

Our retrospective cohort study has several strengths: The German Disease Analyzer (DA) is a large European outpatient database containing data from 2898 practices with about 7.8 million patients in Germany and the representativeness of the diagnoses it contains has already been validated [18, 39]. Furthermore, DA provides continuously updated data generated directly from practice computers based on patient data (diagnoses, demographic data, prescriptions, several measurements, etc.) and has been successfully used for several pharmacoepidemiology studies in various disciplines [18, 40–42]. However, the DA does not contain information on external confounding factors (alcohol and tobacco consumption, socioeconomic status, etc.) and there is also a lack of hospital data and information on mortality that should be considered. Furthermore, diagnoses are based solely on ICD-10 codes documented by general practitioners and do not include diagnoses by gynecologists or urologists, which could influence the quality of the documented diagnoses. Moreover, using ICD-10 Code N39.0 (urinary tract infections, site not specified) is not sufficient to distinguish between upper or lower UTI. In addition, there is a lack of information regarding the specific UTI bacterial spectrum or the exact classification (complicated vs. uncomplicated).

Finally, it should be mentioned that the retrospective cohort design does not allow conclusions to be drawn concerning the causality of the present findings.

## Conclusions

The present study did not identify any significant increase or decrease in UTI incidence or antibiotic therapy in outpatients treated with ACEi, ACB, CCB, beta blockers or diuretics. The incidence of UTI and antibiotic therapy was higher in women than in men across all AH classes, although not significantly.

## Data Availability

Anonymized raw data available on reasonable request.
